# Article 4: Impact assessment of supervision performance assessment and recognition strategy (SPARS) to improve supply chain management in health facilities in Uganda: a national pre and post study

**DOI:** 10.1186/s40545-020-00290-8

**Published:** 2021-02-04

**Authors:** Denis Okidi Ladwar, Moses Nixon Sembatya, Nancy Miriam Amony, Morries Seru, Dennis Ross-Degnan, Laura Garabedian, Birna Trap

**Affiliations:** 1 USAID/Uganda Health Supply Chain Program, Management Sciences for Health, Plot 15, Princess Anne Drive, Bugolobi, P.O. Box 71419, Kampala, Uganda; 2 Pharmacy Division, Ministry of Health Uganda, Plot 6/P.O. Box 7272 Lourdel Rd, Kampala, Uganda; 3 Harvard Pilgrim Health Care Institute, 133 Brookline Avenue, 6th Floor, Boston, MA 02215 USA

**Keywords:** Supportive supervision, Medicines management interventions, Multipronged intervention, Performance assessment, Uganda, Supply chain management, Stock management, Storage management, Ordering and reporting.

## Abstract

**Background:**

To strengthen medicines management capacity, including supply chain management, at public sector health facilities in Uganda, the Ministry of Health introduced a multipronged supervision, performance assessment, and recognition strategy (SPARS). The aim of this study was to assess the impact of SPARS on improving supply chain management. A series of four papers on SPARS described the SPARS concept, its impact on overall and domain practices and appropriate medicines use, and now in the fourth paper describing the effect on supply chain management.

**Methods:**

District-based health workers trained as supervisors build facility-level capacity in medicines management using an indicator-based performance assessment followed by targeted supervisory visits. From 2010 to 2013, 1222 SPARS visits were implemented, and the SPARS performance indicator scores determined. This article assesses impact on 13 indicators in three of the five SPARS domains—stock management, storage management, and ordering and reporting quality—using a pre–post design. We also explored factors that may have influenced these outcomes.

**Results:**

Between the first and last visit within one year of SPARS implementation, we found an average improvement of 16 percentage points (*p* < 0.001) in supply chain management measures across all levels of care. The improvement in scores for stock management, storage management, and ordering and reporting was 22 (ns), 16 (*p* < 0.001), and 11 (*p* = 0.032) percentage points, respectively. The study identified the key predictors of positive change as a low initial indicator score, frequent supervisory visits, and engagement of the district health officer.

**Conclusions:**

The multipronged SPARS approach was effective in building supply chain management capacity in lower-level health care facilities with statistically significant improvements in supply chain management overall and in almost all stock and storage- management and ordering and reporting measures after one year of implementation. We recommend broad dissemination of the SPARS approach as an effective strategy to strengthen supply chain management in low-income countries.

*Trial registration*: The study did not involve or use human participants or identifiable personal data, human tissue, or animals and thus did not require ethical approval or a waiver. It is a study implemented in collaboration with the Ministry of Health and does not require trial registration.

## Background

Essential medicines and health supplies (EMHS) of good quality should be available and accessible at all levels of care to achieve optimal health outcomes [1–3]. To accomplish this, the medicines and commodity supply chain needs to be well-funded and well-managed. Ensuring an effective supply chain involves the management of many stakeholders, systems, complex relationships, and the optimization of processes covering stock management, storage management, and ordering and reporting [4–7].

Though Uganda is committed to ensuring universal access to essential medicines, the health system and the pharmaceutical supply chain have for decades faced many documented constraints, including persistently low availability of EMHS, weak stock and storage management, high rates of product expiry, and an inability to correctly quantify needs or make timely orders [1, 2, 8]. Only 57% of inspected public sector facilities have passed inspection criteria [9], and over half of all health facilities in 2011/12 experienced stock-outs of first-line antimalarials, measles vaccines, oral rehydration salts, and cotrimoxazole [10]. Poorly implemented supply chain management (SCM) can result in significant financial losses, lack of EMHS availability, high rates of expiry, acceleration of drug resistance, and poor health outcomes [11].

In its past attempts to build SCM capacity at facility level, Uganda implemented a number of fragmented educational interventions that did not produce substantial or sustainable improvements in SCM [2, 12, 13]. Poor system capacity in stock management and quantification of supply needs at facility level led to Uganda’s reintroduction of the kit system in 2009 combined with increased EMHS funding for lower-level facilities. This approach initially increased availability but also undermined SCM capacity at lower-level facilities and precipitated further problems in drug expiry and overstocking. The need to find a sustainable strategy and approach to building SCM capacity at all levels of care was evident [14].

Several reviews have documented that improving performance in health facilities is best achieved by combining several approaches, including in-service training and supportive supervision [15–17]. The Ministry of Health in 2010 piloted a novel facility-based capacity-building program known as the supervision performance assessment and recognition strategy (SPARS) [7]. SPARS is implemented by district health workers trained as medicines management supervisors (MMS) who assess performance and supervise at all levels of public health facilities, including those managed by both government and private not-for-profit (PNFP) organizations. The SPARS intervention and its longitudinal impact on overall medicines management and the five underlying domains is described in previous articles of this theme series on improving the pharmaceutical sector in Uganda [7, 18, 19]

A previous study evaluated the overall impact of the SPARS during the first year of supervision in health facilities that entered the program from 2010 to 2013 [18]. Overall SPARS scores (maximum of 25) improved by 2.3 points (22%) per visit from a mean baseline score of 10.3.; the average improvement per visit was highest at the lowest level health facility (2.4 points) and lowest at the highest health care levels (2.2 points). By the end of a year of supervision, 22% of all health facilities had reached an adequate SPARS score of 75% of the maximum 25 points, (i.e., 18.75). The average scores in the three SCM domains out of maximum scores of five in each ranged from 2.9 for storage management, to 2.3 for stock management and 2.2 for ordering and reporting [7].Studies have documented the overall SPARS effect, including on the overarching domains of storage and stock management and ordering and reporting. This study assesses in more detail the impact of the SPARS intervention on individual components and measures within these two domains. To examine the impact of SPARS supervision on specific SCM practices included in the SPARS assessment tool, we examined changes from the initial visit to the last assessment visit that occurred during the first 12 months of supervision in 1222 government and PNFP health facilities at all levels of care in 45 districts.

## Methods

### Design

This study is a pre–post indicator-based comparison of the effect of SPARS on three SCM assessment areas.

### Setting and context

In 2017 the population of Uganda reached close to 38 million increasing from 80 districts in 2010 to 116 districts in 2017 [18]. Health care services are provided through 6404 public and private sector health facilities, of which 3084 (48%) are government owned, 2373 (37%) are private for-profit, and 947 (15%) are PNFP [18]. Uganda’s health facilities are divided into seven levels based on the services they provide and the catchment area they serve. The lowest health center (HC) 1 level comprises village health teams, followed by increasingly larger HC2, HC3, and HC4 health centers; at the highest levels are general or district hospitals, regional referral hospitals, and the two national referral hospitals. Nurses primarily staff HC2; clinical officers and nurses staff HC3; and doctors, clinical officers, nurses, dispensers or pharmacy technicians, and storekeepers staff HC4 and hospitals. Nurses manage medicines at most health facilities because less than 8% of pharmacy posts in the public sector are filled; in general, pharmacists and pharmacy technicians are only available at higher-level facilities [1]. The government-owned National Medical Stores supplies medicines every two months free of charge against a budget allocation for EMHS to all government health facilities using a combination of an order-based supply system for hospitals and HC4 facilities and a kit supply system for HC3 and HC2 facilities. Availability of essential medicines remains low at the National Medical Stores, meeting 56 to 65% of needs [18]. The Joint Medical Store supplies selected EMHS to PNFP facilities using an order-based supply system. Per capita expenditure on essential medicines in the public sector was US$2.40 in 2013/14, of which US$0.99 was for basic essential medicines (up from US$0.50 in 2010/11), and the remaining US$1.41 was for medicines to treat HIV, tuberculosis, and malaria. Funding for EMHS is inadequate and heavily dependent on donors, which covered 77% of essential medicines costs in 2013/14 [1].

### Sampling

To pilot SPARS, we randomly selected 45 districts from the total of 80 districts classified based on their capacity rating of high, medium, and low. Using systematic sampling resulted in 15, 13, 9, and 8 districts representing Western, Eastern, Northern, and Central regions, respectively. In total, we included 1222 facilities that had at least two visits within their first year of implementing SPARS [7]. These facilities were government or PNFP facilities including HC2, HC3, HC4, and hospitals. We stratified our analysis by level of care and grouped HC4 and hospitals together due to the small sample size.

### Data source

We obtained all the data from SPARS supervision visits that had been uploaded by MMS into an electronic database. The SPARS data collection tool is described elsewhere [7] (Additional file 1). SPARS data is organized by 25 indicators covering five domains: stock management, storage management, ordering and reporting, dispensing quality, and prescribing quality. For this study we looked at the SPARS SCM indicators including 35 measures distributed by stock management (3), storage management (28), and ordering and reporting quality (4). The linkage between indicators and measures is in Additional file 2.

The stock management measures are based on a sample of 15 different EMHS. Seven items^1^ were removed from this analysis because they were not required to be stocked at all health facilities, dependent on level of care, facility ownership, and availability of a refrigerator. By removing these seven medicines from the analysis, we assured consistent data that made it possible to compare across all facilities and levels of care. The eight items included in this study were artemether/lumefantrine 20/120 mg (adult dose), amoxicillin 250 mg capsules, benzyl penicillin injection 1 MU, cotrimoxazole 480 mg tablets, oral rehydration salt sachet, sulfadoxine-pyrimethamine 500/25 mg tablets, syringe 5 cc needle disposable 21G, and tetracycline eye ointment.

### Data completeness

To assess change in facility data completeness, we assessed the percentage of facilities with any score at first visit, last visit, both first and last visit, and change in completeness. Completeness was measured as the average completeness of data measures for the first and last visits (Additional file 3). We excluded three (7.9%) measures (i.e., stock book use, order timeliness, and filing of previous orders) from the analysis that had less than 30% data completeness in both first and last visits. On average across the 35 SCM measures, 77% of facilities had a score of 86% at first visit and 82% at last visit.

### Measuring change

We measured the average score across all facilities for each SCM measure between first and last visit in the first year of SPARS calculated as percentage point change. Given that SPARS is intended to provide attainable incentives for improvement, an SCM score of 75% or more following one year of supervision was defined as “adequate” performance. We also calculated the average score of all 35 SCM measures per facility and the proportion of SCM measures scores at first and last visit in three strata of: < 30%, 31–75%, and above 75%, to assess changes in measure scores.

All measures were scored on a binary scale of 1 (yes) or 0 (no) except for four stock management measures that averaged across eight medicines and one order and reporting measure that averaged across six medicines.

The SCM measures were categorized into two categories; 30 (79%) measures that can primarily be improved by behavior change on the part of the facility staff named “behavioral” and 8 (21%) that require primarily resource investments named “resource.” Our analysis compared improvements in the two categories of measures.

The primary outcomes assessed percentage change by overall SCM, domain, individual SCM and category measures, and the percentage of facilities that would reach an adequate average overall SCM score of 75% or more following one year of supervision.

### Predictor variables

The predictor variables that we used have been described in an earlier article [18]. We used two categories of predictor variables—facility and MMS-level predictors. Facility predictor variables were obtained from administrative data or derived from SPARS visit records. MMS variables were obtained from a questionnaire-based survey implemented in 2013 with 111 MMS responding out of the 148 total MMS (75%) who conducted the supervisory visits. We classified the predictors into six groups: initial score, facility type, supervision structure, region, MMS qualifications, and district health officer (DHO) engagement.

Facility-type predictors included: ownership (government or PNFP) and level of care (HC3, HC4, or hospital versus HC2). The supervision structure predictors included the number of SPARS visits in the initial year, the number of health facility staff supervised in the initial MMS visit, if one or two MMS were conducting the supervision visit, and the number of facilities assigned to the MMS conducting the visit. The regions included Central, Eastern, and Northern regions compared to the Western region. The MMS predictors included gender; MMS position (district or sub-district supervisor); professional training (doctor/clinical officer, pharmacist/dispenser, nurse/midwife, or stores officer); highest level of education; and years of work experience. The MMS-related predictor variables also included engagement of the DHO (the frequency of MMS meeting with the DHO or whether the MMS received feedback from the DHO), whether the MMS felt that there was sufficient time to provide adequate supervision, and whether the MMS felt that health care workers responded well to the supervision.

### Data imputation

Based on data from complete cases, we used multiple methods to impute values of missing survey predictors for use in regression models [20, 21].

### Statistical analysis

#### Analysis of change for overall SCM measures, SCM domains, and individual SCM measures

We calculated the number (and percentage) of facilities with any score (i.e., score not missing) for each of the measures at the first visit and the last visit. We restricted analysis of change to measures for which a facility had non-missing data in both first and last visit. For each of the 35 individual measures included in the study, we calculated the percentage of facilities that had a score of 1 (i.e., achieved indicator) at the first visit and at the last visit, and the percentage point change between visits. We calculated two sample tests of proportions (i.e., *Z*-tests) to test for statistically significant differences at first versus last visit for SCM overall scores, domain scores, and indicator category. To avoid type 2 errors due to multiple testing, we used a Bonferroni correction (i.e., alpha = 0.05 divided by number of indicators = 35) to set a conservative, statistically significant *p*-value threshold of 0.001. The overall SCM measures score, domain score, and category of measures score were calculated as averages at first and last visit and as percent point changes between visits and compared across level of care.

#### Predictors of change

To assess the association between each predictor variable and the binary performance measures of interest, we used multivariate logistic regression models. For the three stock management measures and the measure “do health management information system (HMIS) report and stock card agree,” which were comprised of meeting binary performance criteria for multiple drugs, we used a hierarchal generalized linear model, which treated the outcome for each drug within a facility as a repeated measure. We used multivariate logistic models predicting the final scores for outcomes with one score per facility (31 measures). In each model, we controlled for the score at first visit. The models included the facility-level SPARS measures entered into a database by the MMS and MMS survey covariates. We used proc logistics in SAS 9.3 to run the models and we reported the odds ratios and 95% confidence intervals for each indicator. Using multiple testing in our study might have resulted in significance by chance alone, so we therefore highlighted only predictors that affected measures at a significance level of < 0.001.

## Results

### Characteristics of health facilities and visits

MMS visited 1499 facilities between 2010 and 2013 in the 45 sample districts; due to lost or incomplete reports, 1384 facilities (92%) had an analyzable record available for their initial assessment, and 1222 (82%) had at least one follow-up visit in the 12 months after their initial visit and were included in the analysis. Overall, 85% were government and 15% were PNFP facilities, and the analyses included 681 HC2 (56%), 416 HC3 (34%), and 125 HC4 and general hospitals (10%) (Additional file 4).

Facilities were comparable across levels of care by region. Lower-level facilities had higher percentages of government ownership (*p* = 0.002), and fewer facilities had started SPARS supervision in 2011 (*p* < 0.001). At the initial visit, a greater percentage of HC2 were supervised by only one MMS (*p* < 0.001), and higher-level facilities had a greater percentage of initial visits in which two or more health workers were supervised (*p* < 0.001). The designated MMS for a facility conducted the initial supervision in about two-thirds of the facilities.

### Characteristics of medicines management supervisors

Of the 148 MMS included in the study, 84% (124) were male, 64% (95) were health sub-district level MMS, 55% (81) supervised up to 10 facilities, and 59% (87) were trained as clinical officers. Of the 111 MMS that completed the 2013 MMS characteristic survey, 42% (46) were age 36 to 45, 83% (92) had secondary or diploma-level education, and 40% (45) had fewer than 10 years of experience. Most MMS completing the survey reported having a monthly or weekly meeting with the DHO, and 85% (92) received feedback from the DHO on their submitted reports. About two-thirds of MMS felt they had enough time to conduct supervision during visits, and two-thirds thought that health workers responded well to the supervision (Additional file 5).

### Intensity of supervision

In the 1222 health facilities, MMS carried out 4172 supervisory visits in the first year of supervision with an average of 3.4 visits per facility. The median number of visits per facility was 3.0 (interquartile range [IQR] 2–4), and the median number of days between visits was 88 (IQR 61–132). The median number of visits per year per designated MMS was 28 (IQR 17–39) (Additional file 6).

### Change in overall SCM measures score by domain and by category

There was a significant increase of 16 percent points in the overall SCM measures score across all levels of care between the first and last visit. The highest percent point improvement for domain scores was in stock management (22) followed by ordering and reporting (18), and storage management (15). Storage management improvement was significant across all levels of care.

Only behavioral measures improved significantly at all levels of care, with, on average, an 18 percentage point change. Behavioral measures averaged 58% at the start and ended at 76% compared to resource measures that averaged slightly higher at 61% and ended at 71%. Table 1 below shows the change in domain and overall behavioral and resource SCM measures scores.

**Table 1:**
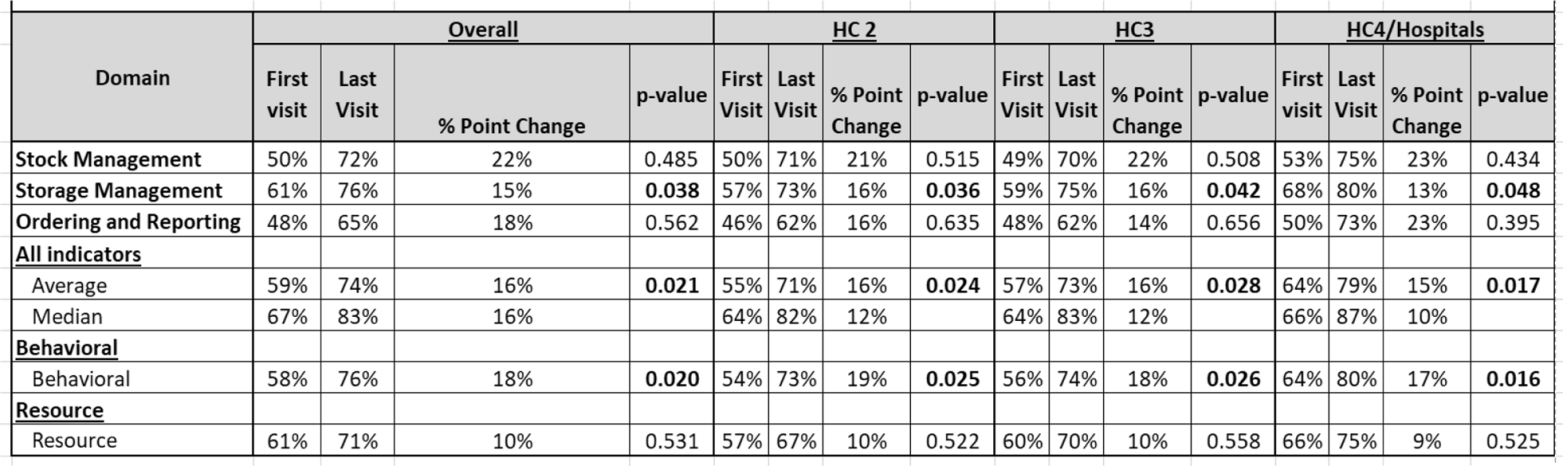
Percentage point change in average supply chain management measures between first and last visit within one year

### Achievement of adequate SCM performance

**Fig. 1:**
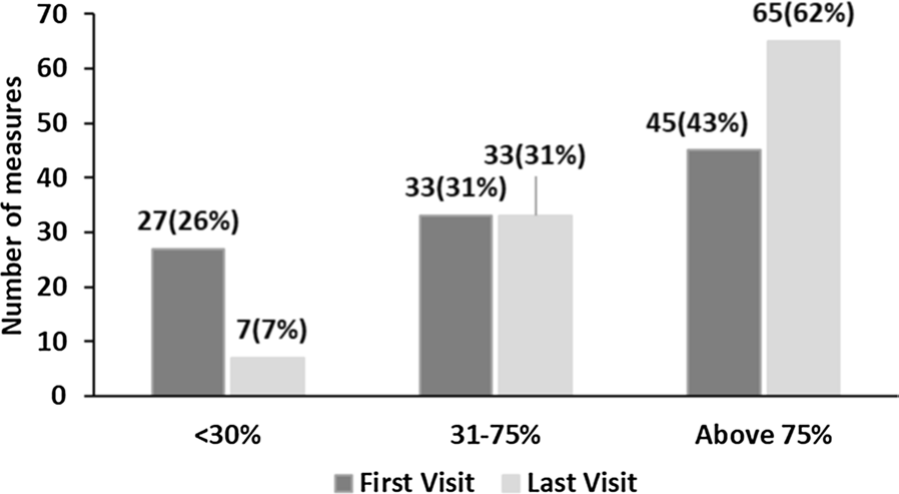
Average scores for the 35 measures calculated across all facilities at first visit and last visit within one year of supervision (*n* = 1222)

**Fig. 2:**
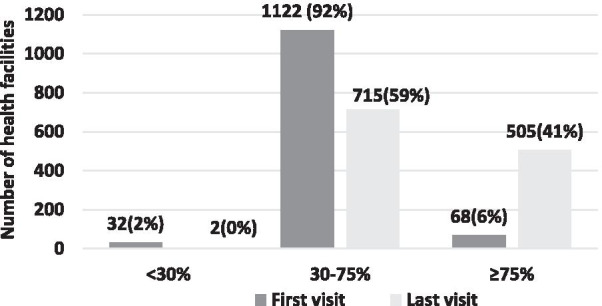
Number of facilities by percentage of all supply chain management measures at first and last visit (*n* = 1222)

The distribution of average scores for the 35 SCM measures for the first and last visit is in Figs. 1 and 2. The proportion of SCM measures reaching an acceptable score of above 75% improved from 43% at visit 1 to 62% at the last visit within one year of supervision (*n* = 1222) (Fig. 1). A greater proportion of HC4/hospitals had more SCM measures reaching an acceptable score (71%) than HC3 and HC2 (57%) at the last visit. At the first visit, only 6% of facilities achieved an acceptable average score on all measures of 75% and above, which increased to 41% of facilities after one year (Fig. 2).

### Impact on individual SCM measure scores

The majority of the individual SCM measures improved significantly at HC2 (29 of 35 measures—83%) and HC3 (28 of 35 measures—80%) compared to HC4 and hospitals (16 of 35 measures—40%) as shown in Table 2.

**Table 2:**
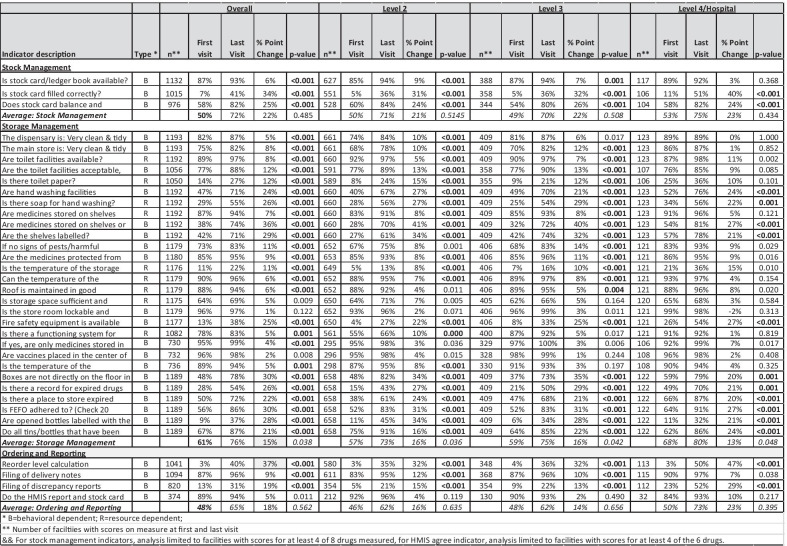
Average percentage point change in supply chain management measures: individually and by category and level of care

### Impact on resource and behavioral measures

The majority of SCM resource measures (availability of stock card, availability of toilets, room temperature monitored, store roof appropriate, medicines stores medicines being stored in refrigerator, storeroom being lockable) already had high scores at visit 1 (all over 85%), which limited improvement by only 2 to 10 percentage points. Most of the SCM behavioral indicator scores on the other hand (reorder level calculation, medicine stored on the shelves in a systematic manner, correct filling of stock card, labeling of shelves) started lower and showed more improvement—on average over 30%.

**Fig. 3:**
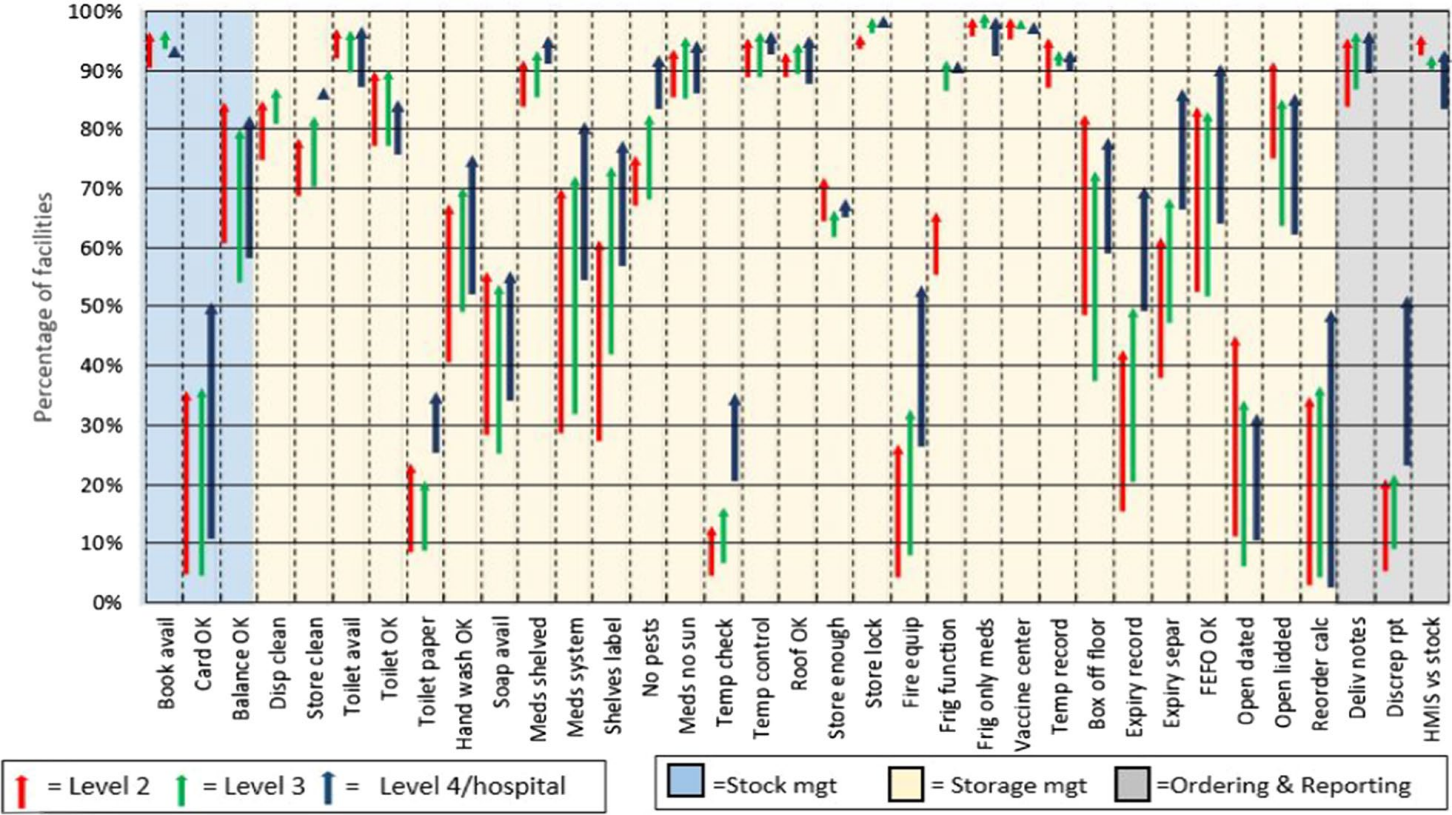
Change in SCM indicator scores at first and last visit in one year of supervision and by level of care

See Fig. 3 below and Table 2 for details.

### Impact on stock management

All three measures in the stock management domain improved significantly across all levels of care. Correct filling of stock cards showed the largest average improvement of 34 percentage points, with hospitals having the biggest improvement of 40 percentage points. Stock card availability averaged over 85% across all levels at the start, so could only improve minimally (Table 2).

### Impact on storage management

The overall improvement of storage management was the lowest of the three domains at 15 percentage points, 13 percentage points HC4 and hospitals and similar improvements at HC2 and HC3 (16 percentage points). The systematic arrangement of medicines on shelves or in cupboards experienced a 36 percentage point change overall with 41, 40, and 27 percentage point improvements at HC2, HC3, and HC4/hospitals, respectively. HC4/hospitals had better practices at the last visit compared to HC2 and HC3 in labeling of shelves, recording of expiry, segregation of expired medicines, monitoring room temperatures and having appropriate cold storage facilities. HC2 and HC3 however were better at last visit in labeling their medicines bottles with opening dates to track how long they stay in the dispensary compared to hospitals. See Fig. 3 below and Table 2 for details.

### Impact in ordering and reporting

Overall, three of four measures in the ordering and reporting domain showed significant improvements. Reorder level calculation knowledge showed the highest overall improvement of 37 percentage points with better improvements in HC4/hospitals compared to lower-level facilities. The second largest improvement was found in the indicator measuring the practice of filing discrepancy reports with 19 percentage points improvement overall; HC4/hospitals’ increase was almost double that of HC2 and HC3 facilities. Over half of all facilities filed discrepancy reports. Improvement *in filing of delivery notes and HMIS report and stock card agree was* only moderately improved with nine and five percentage points, respectively. See Fig. 3 and Table 2 for details.

### Predictors of change

We analyzed the predictors of improvement (i.e., final score adjusted for initial score) in the 31 SPARS measures that were scored as binary outcomes at each visit (Table 3 part 1, Table 4 part 2). Table 5 shows the three stock management measures and the one ordering and reporting measure with more than one score per facility Notable (*p* < 0.001) variations in improvement by key predictors included the following areas (results reported as odds ratios).

**Table 3:**
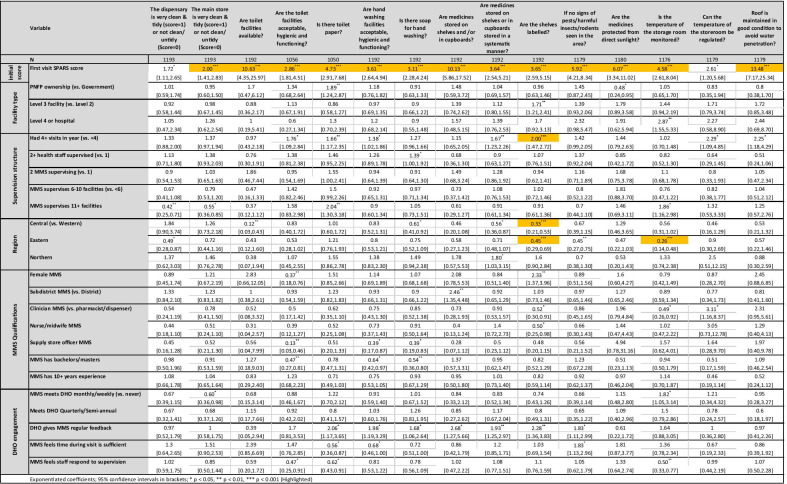
Part 1: predictors from multivariate logistic regression models associated with SCM measures scored as yes/no at each facility

**Table 4:**
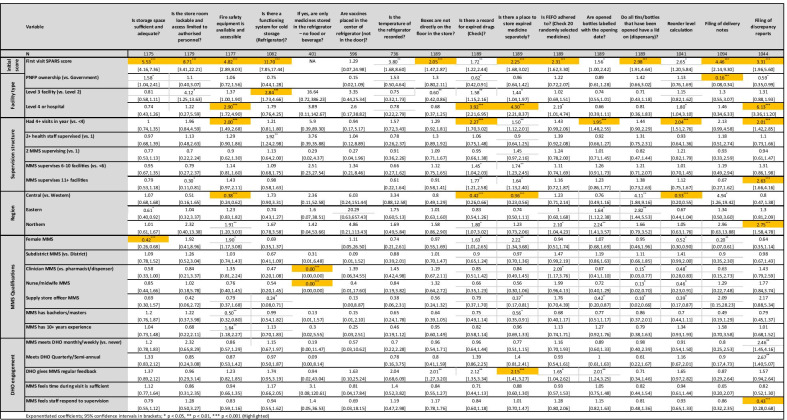
Part 2: predictors from multivariate logistic regression models associated with SCM measures scored as yes/no at each facility

**Table 5:**
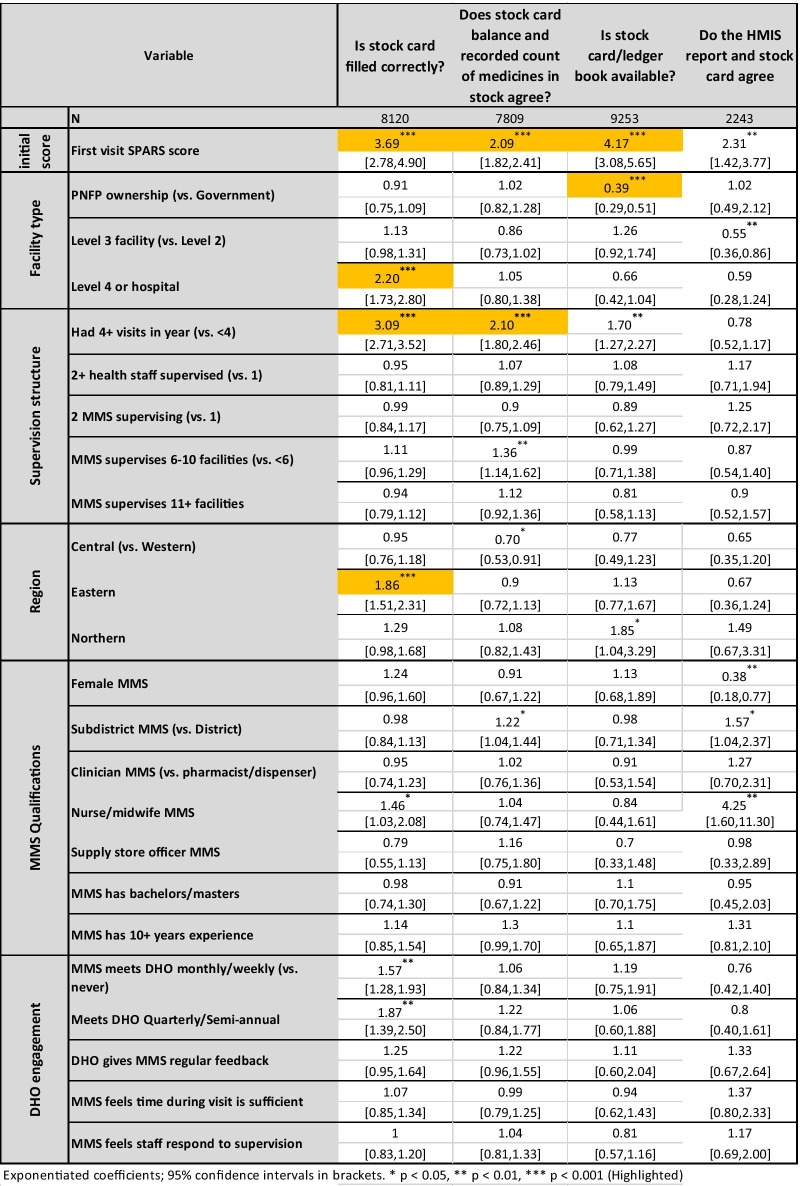
Predictors from multivariate hierarchical logistic regression models associated with SCM measures scored for multiple medications per facility

*Score of initial SPARS visit* All SCM measure increases were significantly associated with the initial SPARS scores with the exception of vaccines placed in the center of refrigerator. This means that measures that started low improved greatly, while measures that started high could only achieve limited improvement between first and last visits.

*Facility type* Several SCM measures improved significantly more at higher-level facilities (HC4/hospitals) compared to HC2 following SPARS supervision including having a record for expired medicines (odds ratio = 3.92, 95% confidence interval = 2.21–6.95), storing expired medicines separately (4.30, 2.21–8.37), filing discrepancy reports on supplies (6.13, 3.36–11.20), using stock cards correctly (2.20, 1.73–2.80), and fire safety equipment is available and assessable (2.90, 1.72–4.90). More HC3 experienced increases than HC2 on having a functioning cold storage (i.e., refrigerator) following supervision (2.84, 1.73–4.66). Government facilities improved significantly following supervision on the use of stock cards compared to PNFP facilities (0.39, 0.29–0.51) and on filling delivery notes (0.16, 0.08–0.34).

*Supervision structure* Having over four supervisory visits per year significantly improved several of the measures including stock cards being filled correctly (3.09, 2.71–3.52), balancing count on card and shelves (2.10, 1.80–2.46), having shelves labeled (2.00, 1.47–2.72), availability of a record for expired medicines (2.27, 1.70–3.02), reorder level calculation (2.04, 1.51–2.76), filing of discrepancy reports (2.01, 1.42–2.85), labeling opened bottles with date of opening (1.95, 1.48–2.55), and availability of fire safety equipment (2.00, 1.49–2.68). Filing of discrepancy report also improved significantly for MMS who supervised more than 11 facilities (2.63, 1.66–4.16).

*Region* Facilities in the Western region experienced significant increases on a number of measures following supervision compared to the Central region, including the availability of record for expired medicines (0.42, 0.26–0.66), storing expired medicines separately (0.36, 0.23–0.56), having labeled shelves (0.33, 0.21–0.53), calculation of reorder level (0.33, 0.20–0.55), and availability of fire safety equipment (0.38, 0.24–0.62). The Western region facilities also improved more than those in the Eastern region on labeling of shelves (0.45, 0.29–0.69) and monitoring temperature in the storeroom (0.26, 0.14–0.48). The Northern region facilities improved more on filing of discrepancy reports (2.75, 1.58–4.7), and the Eastern Region improved more than other regions in filling in stock cards correctly (1.86, 1.51–2.31).

*MMS qualifications* One SCM measure was influenced by the MMS qualification—storing medicines in the refrigerator without any food improved when the MMS was trained as a clinician, nurse, or midwife compared to a pharmaceutically trained MMS (0.00, 0.00–0.00).

*DHO engagement* When the DHO provided feedback to the MMS, storing expired medicines separately improved significantly (2.15, 1.41–3.27), while filing of discrepancy reports improved significantly when the MMS felt the staff members were not responding to supervision (0.43, 0.28–0.68).

The analysis of predictors of change in SCM from first to last visit is in Tables 3 part 1, 4 part 2 and 5.

## Discussion

Following one year of supportive supervision, we found an overall average increase of 16 percentage points in the SCM measures studied, representing a relative improvement of 27% from the initial visit. The levels of improvement at all levels of care in both government and PNFP sectors are greater than the average levels observed in many other interventions. For example, Alex Rowe and colleagues (2018) in a meta-analysis found that supervision combined with other management techniques including training improved health worker SCM performance by 11% [22]. Assuming an initial SCM performance of about 60% and a SPARS effect of 16 percentage points, post-intervention performance would be 76% (i.e., 60 plus 16 percentage points); however, that means that the SCM performance of about a quarter of all facilities would be inadequate, thus jeopardizing patient care through EMHS unavailability, expiry, deteriorated quality, and other safety risks.

SPARS’s impact on SCM and appropriate medicines use was almost the same—16 versus 17 percent points respectively, but slightly lower compared to a 24 percentage point impact on dispensing practices [19]. The larger effect on dispensing practices compared to SCM might be linked to the difference in the initial scores, with the SCM starting point at 65% versus 44% for dispensing, which left more room for improvement [19].

The overall impact of SPARS was almost the same at all levels of care, although with great variation between individual measures. A larger proportion of SCM measures reached the acceptable score of at least 75% at HC4/hospitals than at the lower levels. This could also be associated with availability of dedicated stores assistants or pharmaceutical staff to manage the process at that level [15].

Behavioral indicators improved significantly more than resource indicators, with behavioral indicators starting lower and ending higher. Resource indicators rely—obviously—on resources such as infrastructure that are generally more challenging to influence, thus making it more difficult to improve scores. We found that resources were mobilized from the facility, the community, the Ministry of Health, and from the implementing partners, so all resource indicators did improve; however, we found it easier to change and improve health staff behavior and practices.

### Stock management

The government, PNFP warehouse, and SPARS implementing partners provide stock cards to the facilities, so the availability measure was high at the start; however, the stock cards’ correct use measure was dramatically poor at all levels of health care facilities. Supervision, therefore, focused on ensuring correct use: stock on the shelf tallying with the stock noted on the card and stock cards correctly and completely filled out with a variety of information such as name, strength, average monthly consumption, etc. Staff’s ability to calculate the average monthly consumption was limited, resulting in a score below 50% in this measure, while the majority of cards had the correct stock balance recorded. As a result of supervision, the stock management measure greatly improved by 22% points, and in fact, saw the highest improvement in any of the three SCM domains. As a comparison, in a Zimbabwe study, supervision alone, without the multipronged approach, produced an increase of only seven percentage points [23].

### Storage management

Improvement in storage management was similar across all three levels of care with a slightly lower change at HC4/hospitals due to a higher initial performance score. The storage management domain has the most measures, with one-third of them falling into the resource classification. The improvements across storage management measures differed considerably from 1 to 36 percentage points. Measures with increases of 5% points or below all had an initial score of about 80% or above, which allowed for little change; for instance, two measures that improved only 1% and 2% initially scored 96%:

- If the store room is lockable and access limited to authorized personnel measures is a standard practice in Uganda.
- If vaccines placed in the center of refrigerator (not in the door) is a practice prioritized by the immunization program.

In addition, two resource indicators had limited improvement in spite of low or medium initial scores:

- Storage space is sufficient and adequate which would require community or donor involvement to fund a health facility renovation to increase storage space. In some cases, the facility could identify an extra room but the majority of them could not find a solution, which resulted in 3 to 7% point improvements.
- Is the temperature of the storage room monitored required not only a thermometer but also a new practice of monitoring and recording the temperature. Moreover, and most importantly, very few facilities would even be able to react on this information by regulating the temperature so the motivation for implementing this practice was low.

The indicators that had the highest improvement, including three with 30 percentage points or more, did not have a very low score initially. Two of these indicators were boxes are not directly on the floor in the store and medicines stored on shelves or in cupboards stored in a systematic manner. Both indicators greatly benefitted from an SCM implementing partner providing shelving to all facilities. Although they were behavioral indicators, they would have been difficult to improve without shelving. The third indicator, also a behavioral indicator, was adherence to first expiry first out. This practice required considerable staff capacity building to implement it correctly and much focus with many examples and a role play as part of MMS training.

Though the impact varied greatly between the indicators, it is important to note that the average score of all measures, which indicated correct storage management practices, had increased to 76 percent following one year of supervision, exceeding the 54% seen in another study that assessed storage management [24].

### Ordering and reporting

Because lower-level facilities (HC2 and HC3) receive the majority of their supplies in standard kits, they generally do not have the opportunity to carry out EMHS quantification and ordering; therefore, we excluded related indicators from the study because they are not measured in all facilities. That left only four behavioral indicators for this domain. Overall, following one year of supervision, ordering and reporting improved by 18 percentage points, which was the second highest improvement of the three areas. HC4/hospitals experienced higher increases, perhaps because they have dedicated storekeeping or pharmaceutical staff at that level to focus on SCM.

Two indicators experienced improvements below 10% points, but following supervision, scored 94% and 96%, thus leaving little room for further improvement. Most facilities were routinely filing delivery notes, especially HC4/hospitals, and after one year of supervision, all levels of care had similar scores. On the other hand, HC4/hospitals initially scored more poorly in correctly reporting stock status in the HMIS. After 2 to 10 percentage point improvements in those facilities, all levels of care scored almost equally after one year. Correct reporting in the HMIS in HC4/hospitals was closely linked to their advancements in filling in stock cards correctly, which increased by 40% points.

At baseline, HC2 and HC3 scored similarly low in filing of discrepancy reports, and both levels eventually reached scores of just above 20%. However, higher levels of care were filing discrepancy reports better than lower levels at the beginning, and following supervision reached scores of over 50%. The fact that HC4/hospitals were more likely to file discrepancy reports probably relates to the greater likelihood of mistakes with individual facility orders versus standard kits; in addition, facilities are motivated to file reports because the warehouses will correct the discrepancy.

At close to 40% points, reorder level calculation was the most improved measure. This practice also required extensive work with staff for them to be able to do it correctly, which similarly involved a focus in MMS training and examination for them to teach it effectively. Calculating reorder levels necessitates a good understanding of the calculation itself as well as good arithmetic skills. The indicator only reached an average score of 40% following one year of supervision, however, only 3% were able to calculate reorder level correctly at baseline.

### Predictors of change

We found that the predictors of positive change were a low initial indicator score, frequent supervisory visits, and using the SPARS approach, which align with other studies’ results. A Cochrane review of 49 audit and feedback interventions found that impact is greater when initial performance is low and supervisory visits occur at least monthly [25]. The study also concluded that highest effect was seen when a “supervisor or a senior colleague” provided feedback using both “communicating and writing” with “explicit goals and specific action plan,” similar to SPARS where MMS’s approach is hands-on supportive supervision, and next steps with target-setting are tracked in a supervisory book.

*Facility type* Government facilities improved more on two measures: the availability and use of stock cards and filing of discrepancy reports. These two tools are critical to document stock losses and to minimize thefts. The government uses stock cards as an audit requirement, so managers prioritized their correct use and verified their availability annually. Moreover, most PNFP facilities struggle for funds, and because they had to purchase stock cards from the Joint Medical Store, other purchases came first. Some facilities did improvise with self-made stock cards until the SPARS MMS provided them.

Similar to other studies, we found that intervention effects did vary by level of care [15]^26^, although SPARS supervision did significantly improve performance across all levels of care despite differences in service complexity, resource availability, and staffing. In addition to results discussed above, we found that HC2/HC3 faltered more in practices related to receiving standard kits, such as handling expired medicines, whereas, higher-level facilities quantify and order EMHS to better manage and limit expired medicines. The ability to influence something such as expiry is a motivation to keep records as noted previously with filing discrepancy reports. In addition, high-level facilities have access to more funding making them better able to make infrastructural changes; for example, more than twice as many HC4/hospitals as HC2/HC3 had adequate fire safety equipment after the intervention.

*Supervision structure* Four or more supervisory visits produced better improvement in many of the stock and storage management indicators, which requires behavioral change but also enhanced understanding of why and how the specific practice is to be implemented. Frequent visits with checks and follow-ups gave the health staff a full grasp of the task. Although we have documented that the initial visit had the most impact on practices, subsequent visits continued to have an effect as well [18].

*Region* All regions apart except for the Central region excelled in different measures. This discrepancy may link to SPARS’s phased roll out starting with the Central region. Initially, limited attention was given to the MMS selection process and often the MMS was selected from among focal persons for other Ministry of Health programs. Districts from regions that rolled out SPARS later had stricter guidelines in place for choosing MMS; additionally, DHOs provided input on a candidate’s level of interest and demonstrated supervisory skills [27].

*MMS qualifications* One would expect that MMS trained in pharmaceuticals (i.e., stores managers, pharmacy technicians, and pharmacists) would be best-prepared to understand and supervise SCM practices than MMS with a clinical background [27]. However, our study did not confirm that assumption as the MMS’s educational background significantly influenced only one measure—facilities supervised by a clinically trained MMS observed greater improvements in storing only medicines and not food or drinks in the refrigerator. We are not clear on the explanation for this, but perhaps an MMS with clinical training has more knowledge about why this practice would be dangerous.

*DHO engagement* DHO engagement significantly influenced the facilities. Facilities supervised by MMS who received DHO feedback adhered better to storing expired medicines separately (*p* < 0.001). Having regular meetings and feedback from the DHO were associated (*p* < 0.01) with improved performance in seven others measures. Having a DHO interested in your work and performance is inspiring and can make a substantial difference in the achievements observed [15, 18, 27]. Meaningful engagement with the DHO is therefore critical for sustaining SCM improvements in facilities following support visits.

### Study limitations

Our randomized sampling of about half of Uganda’s districts from all regions ensured a diverse range of poverty, district capacity, and performance and provides a good cross-sectional representation of Uganda [7]. However, facilities were not randomized but instead chosen by the MMS, which may constitute a possible bias if not all facilities in the district were included. The MMS eventually covered all facilities in their district, but the order and speed of coverage varied depending on facility ownership, level of care, needs, and proximity as well as DHO directives and, very importantly, MMS workload, time allocated to supervise, and motivation. The supervised facilities represented about a third of all government and PNFP facilities. PNFP facilities comprised 15% of the sample, which is a slight underweighting compared to the actual figure of 23% [28]. The sample’s proportions of HC4, HC3, and HC2 varied somewhat from actual figures, but we did not control for it as it was determined by the district and MMS; however, we did analyze for differences linked to level of care.

The donor-funded implementing partner provided all MMS the same support that enabled them to implement SPARS uniformly, such as motorbikes, riding gear, fuel, computers, tools, training etc. Nevertheless, the MMS had different educational backgrounds, supervisory experience, and interest as well as different levels of support from district management and the DHO. Being a field study, we did not try to control for these factors, but they likely influenced impact [27].

We followed each facility for one year from the date of its initial SPARS visit. The number of supervisory visits per facility ranged from two to seven; about 300 facilities had 2, 3, or 4 visits and slightly less had 5 or more visits within the 12 months, averaging 3.4 visits per facility in the first year (Additional file 6). All MMS had equal access to transport, fuel, and other resources that could constitute a barrier to facility visits. The number of facility visits thus depended on the MMS’ workload (number of facilities allocated to the MMS), dedication, and time available to carry out the SPARS role in addition to his or her regular job responsibilities. Adding SPARS to the ordinary MMS’s roles may have considerably influenced the number of monthly supervisory visits and therefore improvements seen within a year. The number of visits for an MMS ranged from 17 to 39, a difference of 100%, with a median of 28 visits per year. As SPARS was rolled out, some districts also took steps to release MMS from other services and create posts for district or provincial pharmacists’ positions to manage SPARS implementation.

An important limitation in the study design is the lack of a control group. First, we found it would not be financially feasible to establish a control group of comparable size, and second, as SPARS became a national strategy, districts were continuously enrolled. However, we believe that the consistent improvements in SCM performance that we observed are likely due more to the intervention rather than to unobserved factors as we documented in a longitudinal study [18]

The study also did not stratify for score of measures at visit 1 to ensure that change was measured from a near similar situation at baseline due to the large number of measures.

The MMS predictors of change were based on an MMS survey with a 75% response rate despite several follow-ups. However, results from using multiple methods to impute values of missing survey predictors to use in regression models were basically the same as using only cases with complete data.

The year a facility began SPARS did not measurably influence impact, although facilities that joined later in the study period likely knew about SPARS prior to their first visit. Without a control group, we cannot rule out the possibility that some contamination from earlier SPARS facilities and improvements to implementation over time may have led to a slight improvement in SCM in all facilities in the district.

## Conclusion

Building SCM capacity at health facilities at all level of care and in all sectors is fundamental to optimize the use of limited resources and ensure the availability of good quality, life-saving medical products that improve patient-centered care. This study demonstrates that within one year, supportive supervision combined with managerial interventions, performance monitoring, and achievement recognition effectively improved stock and storage management and ordering and reporting in the low-resource health care setting of Uganda.

Despite the overall significant improvement in performance impact differed considerably and there is a need based on our findings of influencing factors to optimize the impact of the SPARS intervention.

Improving facility-level SCM to ensure the availability of quality medicines is as relevant as it was in 2010 when SPARS was launched [29] and SPARS is still relevant to many countries struggling with SCM issues and now also the corona virus putting extra demand on health care provision. Compared with other studies of multipronged, supervision-based interventions, the SPARS model outperformed them. The model can be modified to country-specific issues and disease specific issues or supplies, in fact, Uganda has adapted SPARS to build medicines and supply management capacity within laboratory services and tuberculosis and HIV/AIDS programs [30]. We recommend that the SPARS concept be broadly disseminated and tailored to specific country needs with the aim of strengthening supply chain management performance in all health care facilities.

## Supplementary Information

**Additional file 1: **Health facility supervisor’s monitoring and reporting tool.

**Additional file 2:** Supply chain management Assessment Indicators and measures.

**Additional file 3:** Completeness of measures at first and last visit.

**Additional file 4:** Facility and visit characteristics.

**Additional file 5:** Medicines management supervisor and district health officer characteristics.

**Additional file 6:** Number of MMS visits within the first year of SPARS supervision, overall and by level of care.

## Data Availability

The data, data collection tool, interviews, analysis, and other materials are provided either in supplementary files or can be obtained from the corresponding author.

## Abbreviations

DHO: District health officer
EMHS: Essential medicines and health supplies
HC: Health center
MMS: Medicines management supervisors
PNFP: Private not-for-profit
SCM: Supply chain management
SPARS: Supervision, performance assessment, and recognition strategy

## Footnotes

1 Medroxyprogesterone (Depo-Provera) 150 mg/ml inj); ethinylestradiol + levonorgestrol (Microgynon) 30 µg + 150 µg; measles vaccine inj IM/SC; first-line anti-TB drug (RHZE); first-line antiretroviral tablets (adult) (AZT + 3TC + NVP); surgical gloves latex, 7.5; and malaria rapid diagnostic test.
