# Supplementary material for: Article 4: Impact assessment of supervision performance assessment and recognition strategy (SPARS) to improve supply chain management in health facilities in Uganda: a national pre and post study
Source: J Pharm Policy Pract. 2021 Feb 4;14:14. doi: 10.1186/s40545-020-00290-8 (PMC7857862; doi:10.1186/s40545-020-00290-8)
Supplement: Supplementary file 1 — Additional file 1: Health facility supervisor’s monitoring and reporting tool. [file 40545_2020_290_MOESM1_ESM.pdf]

HEALTH FACILITY SUPERVISOR'S MONITORING AND REPORTING TOOL
FOR
STOCK MANAGEMENT, PHARMACY PRACTICES AND RATIONAL DRUG USE

Region:					
District:		Health Sub District:			
Health Facility:		Level:		Ownership:	
Date Of Visit		Supervision Visit No.:			
Date of Next Visit:		Accreditation:			

NAME OF PERSONS SUPERVISED

#	Name	Gender (F/M)	Profession	Contact/Phone No.
1.				
2.				
3.				
4.				

NAME OF SUPERVISORS

#	Name	Contact/Phone No.	Title
1			☐ District MMS **or** ☐ HSD MMS
2			☐ District MMS **or** ☐ HSD MMS
3			

Read and refer to
"Guidelines on how to use the health facility supervisor's monitoring and reporting tool"
or
"Key points to note when using the supervision monitoring and reporting tool"
before filling the form or in case of clarification needs.

I. DISPENSING QUALITY

1. Dispensing Time

Observe and record the dispensing time for 6 patients.

Patient number	1	2	3	4	5	6	Average	Comments
Dispensing time in seconds								

Score: average <30 seconds= 0; 31-60 seconds =0.5; > 61 seconds=1: ___________ Percentage: ___________

2. Packaging Material

Observe and verify the packaging material available and in use (Yes=1/No=0)

		1/0	Comments
a)	Are appropriate dispensing envelopes available?		
b)	Are appropriate clean containers available i.e. bottles made specifically for the purpose of dispensing liquids		
	Sum		

Score: the sum of a) to b) yes (1) divided by 2: ___________ Percentage: ___________

3. Dispensing equipment

Verify that the dispensary has the following equipment in the dispensing area (Yes=1/No=0)

Equipment	1/0	Comments
a) A spatula or spoon		
b) Tablet counting tray or similar		
c) Tablets not counted by bare hands		
d) Graduated measuring cylinder		
Sum		

Score: the sum of yes (1) of a) to e) divided by 4: ___________ Percentage: ___________

4. Services available at the dispensing area

Verify that the dispensing area/health facility has the following services (Yes=1/No=0)

Services	1/0	Comments
a) Chairs or bench to sit on in dispensing area		
b) Can privacy be achieved during dispensing?		
c) Facility to wash hands by patient in facility?		
d) Drinking water (to take tablets) by patient in dispensing area?		
Sum		

Score: the sum of yes (1), a and b divided by 4:___________ Percentage: ___________

5. Patient care

Interview 5 patients and ask to see the medicines they have received and if possible their prescription. Select one of the medicines to check patient knowledge

	**No. of medicines**		**Patient knowledge** (Yes=1/No=0)					
**Pt no.**	**Prescribed**	**Dispensed**	**Discrepancy (Y=1/ N=0)**	**Dose/ How much to take**	**Freq/ How often to take**	**Duration/ how long to take**	**Does pt. know why s/he is getting the treatment**	**Other information given: Adverse reactions, etc**
1								
2								
3								
4								
5								
Sum								
% (sum/5)*100			100-[(sum/5)*100]					Sum:
**Average score = Average % score of the 5 columns:**								

Score: if average: **> 90% = 1; 75-89%=0.75; 50-74%=0.5; 30-49%= 0.25; <30% = 0**:_________ Percentage: __________

Comments:

6. Labelling

Interview 5 patients and ask to see the medicines they have received. Select one and check for labelling (Y=1/ N=0)

Medicine no.	Medicine name*	Strength	Quantity	Date	Dose	Patient name	Facility name
1							
2							
3							
4							
5							
Sum							
% (sum/5)*100							
**Average score = Average % score of 7 columns:**							

**Note:* *The medicine name appears by generic name or by brand* *and* *generic name*

Score: if average score: **≥ 90% = 1; 75-89% = 0.75; 50-74% = 0.5; 30-49% = 0.25%; < 30% = 0**: ________ Percentage: _________

Comments:

7. Rationing of Antibiotics

Select the last 5 entries including amoxicillin and the last 5 entries including cotrimoxazole from the prescription and dispensing log and record the amount of amoxicillin and cotrimoxazole prescribed and that dispensed, respectively. If out of stock include in your comments, Write **"NA" for discrepancy** and go to Cotrimoxazole or next question

**Drug**	**Pt. no.**	**1**	**2**	**3**	**4**	**5**	**Sum**	**Score: [1 – (sum/5)]**	**Percentage**
**Amoxicillin**	Amount prescribed						Comment:		
	Amount dispensed								
	Discrepancy (Y=1/N=0)								
**Cotrimoxazole**	Amount prescribed						Comment:		
	Amount dispensed								
	Discrepancy (Y=1/N=0)								
Average score and percentage									

Score: (The sum of score of Amoxicillin + Cotrimoxazole)/2: ___________ Percentage: ___________

II. PRESCRIBING QUALITY

8. Correct use of prescription recording system

Record from the last 10 entries from the prescription (or OPD) and dispensing log and note whether the information below have been entered/recorded (Yes=1/No=0)

Prescription entries	Date#	OPD/IP No.	Diagnosis+	Medicines name*	Prescriber's name	Amount prescribed	Amount dispensed	Sum	% (sum/7) *100
1									
2									
3									
4									
5									
6									
7									
8									
9									
10									
Sum									
% Average = % Sum of Last column/10									

# Date should be date of visit, date of previous day or last date the facility received patients if the previous day was a weekend

+Diagnosis refers to definite diagnosis e.g. Malaria, URTI, not symptoms e.g. fever **Brand and generic name or generic name alone.*

Score: if % Average: a) 100% = 1; b) 75-99% = 0.5; c) <75% = 0: ___________ Percentage: ___________

Comment:

9. Rational prescribing

Systematically select 20 prescriptions from past 2 months and record information in table below (Yes=1/No=0):

*****_When carrying out routine supervision randomly select the 20 prescriptions from the last date of supervision_

Pt No.	No. of medicines	No. of medicines prescribed by generic name	No. of antibiotics prescribed	No. of injectables prescribed	No. & name of medicines not in the EMHSLU	Diagnosis recorded Diagnosis=1/ symptoms=0
1						
2						
3						
4						
5						
6						
7						
8						
9						
10						
11						
12						
13						
14						
15						
16						
17						
18						
19						
20						
**Total**	-------------	-------------	# Patient/Drugs -----------/-----------	# Patient/Drugs ------------/-----------	----------------	# Diagnosis ----------------
**Average**	(total/20) -------------		% Patients receiving 1 or more antibiotics ----------------	% Patients receiving 1 or more injection. ------------------		(D/total i.e. 20)*100 ------------------
**%**		% of medicines prescribed by generic name ---------------	% of drugs being antibiotics ------------------	% of drugs being injections ------------------	% of drugs not in the EMLU ------------------	

Transfer calculated results to table below and score accordingly.

Parameter	% or value	How to score	Score
1. Average no. of medicines prescribed per patient		<u><</u>2.5: score 0.2 else 0	
2. % of medicines prescribed by generic name		<u>></u>85%: 0.2 else 0	
3. % of patients prescribed 1 or more antibiotics		<u><</u>15%: 0.2 else 0	
4. % of patients prescribed 1 or more injections		<u><</u>15%: 0.2 else 0	
5. % Diagnosis recorded		<u>></u>85%: 0.2 else 0	
		Sum of the 5:	

Score: sum of the 5 above: ___________ Percentage: ___________

Comments:

Adherence to standard treatment guidelines (for page 5 and 6)

Record from the prescriptions the adherence to treatment guidelines for diarrhoea, non-pneumonia respiratory tract infection (cough/cold) and uncomplicated malaria. For each condition, sample the last 10 prescriptions from the OPD book for each diagnosis. Only consider prescriptions from the last month. If there are not 10 prescriptions with that diagnosis in the last month, leave the remaining fields blank and write comment. **Only give score if more than three cases**

10. Diarrhoea (No blood)

The appropriate diagnosis is diarrhoea. Only look at prescriptions for the last month from today's date.

Disease/Drug prescribed		Cases (Yes=1, No=0)										
**Diarrhoea (No blood)**		1	2	3	4	5	6	7	8	9	10	
1	ORS											
2	Antibiotics											
3	Anti-diarrhoeal											
4	Zinc											
5	Vitamin A											
6	Anti-spasmodic											
7	Albendazole/Mebendazole											
8	Other drugs given											Sum of A=
Assessment (A=1,B=0)												%A(1) = ((sum/total)*100)

Assessment: A is given (if ORS = 1, Zinc = 1 or 0, Vitamin A = 1 or 0, Albendazole/Mebendazole = 1 or 0) or **ELSE** 0

Score: If %1's ≥ 50 then score =1, else 0: __________ Percentage: ___________

Comments:

11. Cough/Cold

The appropriate diagnosis for this are: Non-pneumonia, ART, ARTI, common cold, flu, cough, cold, sore throat.

Disease/Drug prescribed		Cases (Yes=1, No=0)										
**Cough/cold**		1	2	3	4	5	6	7	8	9	10	
1	Antibiotics											
2	Antipyretic/ analgesic											
3	Cough or cold drugs											
4	Albendazole/Mebendazole											
5	Other drugs given											Sum of A=
Assessment (A=1, B=0)												%A (1) = ((sum/10)*100)

Assessment: A, is given (if antibiotics = 0 (No antibiotic given), Antipyretic/analgesic = 0 or 1 and/or Cough/Cold drugs 0 or 1; Albendazole/Mebendazole = 1 or 0] or **ELSE** 0

Score: If %A (1) ≥ 50 then score =1, else 0: __________ Percentage: ___________

Comments:

12. Malaria treatment (uncomplicated malaria)

Consider both malaria and clinical malaria cases**.** In case **no** RDT for malaria or functioning laboratory is available, **score 'Rapid test or smear conducted' with NA.**

Availability of testing facilities

Are Rapid Diagnostic Tests for malaria available? ☐ Yes or ☐ No

Is there a functional laboratory at the facility? ☐ Yes or ☐ No

	**Testing** (1/0)	1	2	3	4	5	6	7	8	9	10	Sum	%(sum/10)*100
1	Rapid test or smear conducted												
	**Treatment** (1/0)												
2	Artemether /Lumefantrine (ACT)												
3	Quinine Tab.												
4	Pyrimethamine /Sulfadoxine SP												
5	Antibiotics												
6	Paracetamol/Antipyretic												
7	Albendazole/Mebendazole												
8	Other drugs given												
	***Appropriate treatment (Y=1/N=0)**												
**Assessment (A=1,B=0)**													%A (1) = ---------

**Appropriate treatment Y=1 if ACT given or Quinine tabs (but not both), Paracetamol=0 or 1, Antibiotics=0, SP=0, other drugs=0; else N=0*

Assessment is given: [if Mal test =1 and ACT = 1 or Mal test =1 and Quinine =1 AND Paracetamol=0 or 1, Albendazole/Mebendazole = 1 or 0] or **ELSE** 0

Transfer calculated results to table below and score accordingly.

Parameter	% or value	How to score	Score
1. % Testing		If % Testing is 100%, then score 0.5, else 0	
2. % Appropriate treatment		If = 100%, then score 0.5, else 0	
3. Assessment (A=1, B=0)		If %A $\geq$ 50%, then score 1, else 0	
**Average %** (of the 3 above)			
		**Sum**	
		**Overall score = (sum/2)**	

Comments:

III. STOCK MANAGEMENT

13 – 16 Availability and correct use of stock cards, stock books etc.

Verify information recorded for the selected EMHS and complete the table (Y=1/N=0): If a product is never stocked, write 'NA' in all columns including column 2; if stock card unavailable write '0' in column 3 followed by 'NA' for other columns – only fill in column 2, 3, 13 and 14. If AMC not calculated write 'NR', not recorded. If item overstocked (column 16) for any of the 5 first items indicate by circling the highest balance. Action then needs to be taken. Don't leave blank fields and explain all 'NA'!

	**Name of medicine**	Unit pack (e.g. tin of 1000)	Item available? (check 1/0) Mark if expired (E)	**Stock card/ledger book available (1/0)**	Is physical count done every month and PC marked in stock card (check 3 months) (1/0)	**Is the card filled correct with name, strength, dosage form, AMC, special storage (1/0)**	Balance according to stock card (record no. from the card)	Count the no. of drugs in stock and record	**Does balance & PC agree 100%? (1/0)**	Record their monthly consumption (AMC) NR/No.	Record the amount issued in the last 3 months (From day of survey)	Record the number of days out of stock in the last 3 months (day of survey)	Calculated AMC (only calculate for the first 5 items)	Is stock book in use (with entry each month each drug)	**Is stock book correctly used (all fields filled & AMC)**	No. of days out of stock for the last 6 months; record no. of days (from day of survey)	Record the highest balance on hand in the last 6 months (from day of survey). Circle if overstocked for first 5 items (calculated AMCx5)
1	ACT Artemether/Lumefantrine 20/120mg (Adult dose) [T]	Pack of 30 blisters															
2	Amoxicillin 250mg caps	Tin of 1000															
3	Benzyl penicillin inj 1 MU	Vial															
4	[¥] Cotrimoxazole 480mg tab [T]	Tin of 1000															
5	Medroxyprogesterone (Depo-Provera) 150mg/ml inj [T]	Pack of 25 vials															
6	Measles vaccine inj IM/SC [T]	Vial															
7	Ethinylestradiol + levonorgestrol (Microgynon) 30µg+ 150µg	Cycle															

	Name of medicine	Unit pack (e.g. tin of 1000)	Item available? (check 1/0) Mark if expired (E)	**Stock card/ledger book available (1/0)**	Is physical count done every month and PC marked in stock card (check 3 months) (1/0)	**Is the card filled correct with name, strength, dosage form, AMC, special storage (1/0)**	Balance according to stock card (record no. from the card)	Count the no. of drugs in stock and record	**Does balance & PC agree 100%? (1/0)**	Record their monthly consumption (AMC) NR/No.	Record the amount issued in the last 3 months (From day of survey)	Record the number of days out of stock in the last 3 months (day of survey)	Calculated AMC (only calculate for the first 5 items)	Is stock book in use (with entry each month each drug)	**Is stock book correctly used (all fields filled & AMC)**	No. of days out of stock for the last 6 months; record no. of days (from day of survey)	Record the highest balance on hand in the last 6 months (from day of survey). Circle if overstocked for first 5 items (calculated AMCx5)
8	ORS sachet [T]	Pack of 25															
9	Sulphadoxine-Pyrimethamine 500/25mg tab (SP) [T]	Tin of 1000															
10	Syringe 5cc needle disposable 21G	Pack of 100															
11	Tetracycline eye ointment	3.5g tube															
12	1st line anti- TB drug (RHZE)	Pack of 24															
13	1st line ARV tabs (adult) (AZT+3TC+NVP)	Pack of 60															
14	Surgical gloves latex – 7.5	Pair															
15	Malaria Rapid Diagnostic Test	Box of 25															
	**Sum**																

Comments (all 'NA' must to be explained! – if needed continue comments after scoring table on next page):

13 – 16. Availability and correct use of stock cards, stock books etc - continued

Scoring:
Use the sums from the table on the previous 2 pages to calculate the score. Remember to subtract 'NA' from the 15 when calculating the score, e.g. where a product is not stocked by the facility.

Indicator	How to score*	Score	Percentage
**13. Stock card availability** (Column No.3)	Sum/(15-NA)		
**14. Correct filling of stock card** (No.5)	Sum/(15-NA)		
**15. Does physical count agree with stock card balance?** (No.8)	Sum/(15-NA)		
**16. Is Stock book correctly used?** (No.13)	Sum/(15-NA)		

Comments:

IV. STORAGE MANAGEMENT

17. Cleanliness of the pharmacy (dispensary and main store)

(Tick only one	**Very clean & tidy (score=1)**	**Not clean/ untidy (Score=0)**	**Comments**
**The dispensary is:**			
**The Main store is:**			
**Sum**			

Score: the sum of score for dispensary + storage area divided by 2 = ___________ Percentage: ___________

18. Hygiene of the pharmacy

Ask to be shown the toilet and hand washing facilities used by the pharmacy (dispensary & store) staff. If there are no toilets, write **'NA'** only for b) and c)

	**1/0/NA**	**Comments**
a) Are toilet facilities available?		
b) Are the toilet facilities acceptable, hygienic and functioning?		
c) Is there toilet paper?		
d) Are hand washing facilities acceptable, hygienic and functioning?		
e) Is there soap for hand washing?		
**Sum**		

Score: the sum of a) to e) divided by 5 minus any 'NA': ___________ Percentage: ___________

19. System for storage of medicines and supplies

Ask to be shown around the pharmacy (main store) and observe the following conditions

	**1/0**	**Comments**
a) Are medicines stored on shelves and /or in cupboards?		
b) Are medicines stored on shelves or in cupboards stored in a systematic manner (alphabetic, therapeutic, formulations)?		
c) Are the shelves labelled?		
**Sum**		

Score: the sum of a) to c) yes (1) divided by 3:___________ Percentage: ___________

20. Storage conditions

Ask to be shown around the main store and observe the following conditions

		1/0/NA	Comments
a)	If no signs of pests/harmful insects/rodents seen in the area (Check traces, droppings etc from bats, rats, ants, etc)**score** 1 **or else** 0		
b)	Are the medicines protected from direct sunlight (Painted glass, curtains or blinds – or no windows)?		
c)	Is the temperature of the storage room monitored?		
d)	Can the temperature of the storeroom be regulated (Ventilation, heater, air-condition, windows)?		
e)	Roof is maintained in good condition to avoid water penetration?		
f)	Is storage space sufficient and adequate?		
g)	Is the store room lockable and access limited to authorised personnel?		
h)	Fire safety equipment is available and accessible (any items for promotion of fire safety should be considered)		
i)	Is there a functioning system for cold storage (Refrigerator)?		
j)	If yes, are only medicines stored in the refrigerator – no food or beverage?		
k)	Are vaccines placed in the center of refrigerator (not in the door)?		
l)	Is the temperature of the refrigerator recorded?		
	**Sum**		

Score: the sum of a) to l) yes (1) divided by 12 minus NA's: ___________ Percentage: ___________

21. Storage practices of medicines in the pharmacy (stores & dispensary)

		1/ 0	Comments
	**Store**		
a)	Boxes are not directly on the floor in the store?		
b)	Is there a record for expired drugs (Check)?		
c)	Is there a place to store expired medicine separately? (Box clearly marked expired or in a shelf)		
d)	Is FEFO adhered to? (Check 20 randomly selected medicines)		
	**Dispensary**		
e)	Are opened bottles labelled with the opening date?		
f)	Do all tins/bottles that have been opened have a lid on (dispensary)?		
	**Sum**		

Score: the sum of a) to f) yes (1) divided by 6: ___________ Percentage: ___________

V. ORDERING AND REPORTING QUALITY

22. Reorder level calculation

Ask the supervisee how s/he decides the amount to order (*including HC II & III, if they were to order)*

No.	Responses	1/0/NA
a)	Select a stock card and stock book; select one drug (e.g. ACT) and check whether the person knows how to determine the quantity to order. Let the person show you how to calculate the quantity to order of the selected drug **and record figures in spaces below.**<br><br>Record: SOH= ………; Issued out (3 months) =...........; Stock out days (3 months) =..........;<br><br>AMC=…….; Maximum quantity (AMCx5) =…………………..<br><br>(Quantity to order = Maximum stock – Stock on hand)=……………..<br><br>(Write 1 if quantity to order is correct)	
	**score**	

23. Timeliness of Orders and distribution

Complete the dates of orders and delivery in the table below for the last order. The final score is 1 or 0 depending on timeliness of ordering. Overall score for HC II and HC III is NA.

****Fill all rows 1 to 5 for HC IV and hospitals; fill in only rows 3 to 5 for HC II & HC III as they receive kits and for PNFPs write a comment.***

No		Cycle 1	Comments
1	Ordering schedule date		
2	Actual date of ordering by facility (write date stamped by DHO)		
	**Was ordering timely (Y=1/N=0)**		
3	Delivery schedule date		
4	Date of delivery from NMS		
5	Date of delivery from district/HSD		

Score (for timeliness of order): __________ Percentage: __________

24. Accuracy of HMIS report: Check the accuracy of the last **HMIS 105** report (Yes = 1/ No=0)

**Date report was filled (use last report or last but one:........... /.......... /...........**				
**Medicine**	**Is information on stock out days available from the last report (1/0)**	**No. of stock out days for month as recorded on the report (# days, NA)**	**Stock out days for that month on the Stock card (# days, NA)**	**Do the report and stock card data agree? (1/0/NA))**
1. Artemether/ Lumefantrine				
2. Cotrimoxazole tabs				
3. ORS sachets				
4. Measles Vaccine (NA if no refrigerator)				
5. Sulphadoxine/ Pyrimethamine(SP)				
6. Depo-Provera (NA for catholic PNFP)				
			**Sum**	
			**% Accuracy = [Sum/(6 - NA)*100**	

Score: the sum of 1 – 6/6 - NA): __________ Percentage: ___________

Comments:

25. Filing: Are the following filed? Check and see

**No.**	**Item**	**Yes=1/No=0/NA**	Comments
1	Discrepancy reports		
2	Delivery notes		
3	Previous orders made (**not applicable to HC2 or HC3**)		
	**Sum**		

Score: the sum of 1-3 divided by 3minus NA's: __________ Percentage: __________

MEDICINES MANAGEMENT SUPERVISION DASH BOARD

Health Facility:		Health Sub District:	
District:		Supervisor Name:	

Indicators	Score	% or Value
**Form 1:** **Dispensing quality (7)**		
1. Dispensing time		
2. Packaging material		
3. Dispensing equipment		
4. Services available at the dispensing area		
5. Patient care		
6. Labelling		
7. Discrepancy between prescribed & dispensed medicines		
**TOTAL** (Add bullets 1-7)		
**Prescribing quality (5)**		
8. Correct use of prescription recording system		
9. Rational prescribing:		
• Average no. of medicines prescribed per encounter		
• % of medicines prescribed by generic name		
• % of patients prescribed 1 or more antibiotics		
• % of patients prescribed 1 or more injections		
• % Diagnosis recorded		
Adherence to STGs:		
10. Diarrhoea		
11. Cough/cold (simple Respiratory Tract Infection)		
12. Malaria (Average % and overall score)		
**TOTAL** (Add bullets 8-12)		

Indicators	Score	% or Value
**Form 2:** **Stock Management (4)**		
Availability and correct use of stock card and stock book		
13. Stock card availability		
14. Correct filling of stock card		
15. Does physical count agree with stock card balance?		
16. Is stock book correctly used?		
**TOTAL** (Add bullets 13-16)		
**Storage management(5)**		
17. Cleanliness of the pharmacy		
18. Hygiene of the pharmacy		
19. System for storage of medicines		
20. Storage conditions		
21. Storage practices of medicines		
**TOTAL** (Add bullets 17-21)		
**Ordering and reporting quality (4)**		
22. Reorder level calculation		
23. Timelines of Order and distribution		
24. Accuracy of HMIS reports		
25. Filing		
**TOTAL** (Add bullets 22-25)		

SpidoGraph

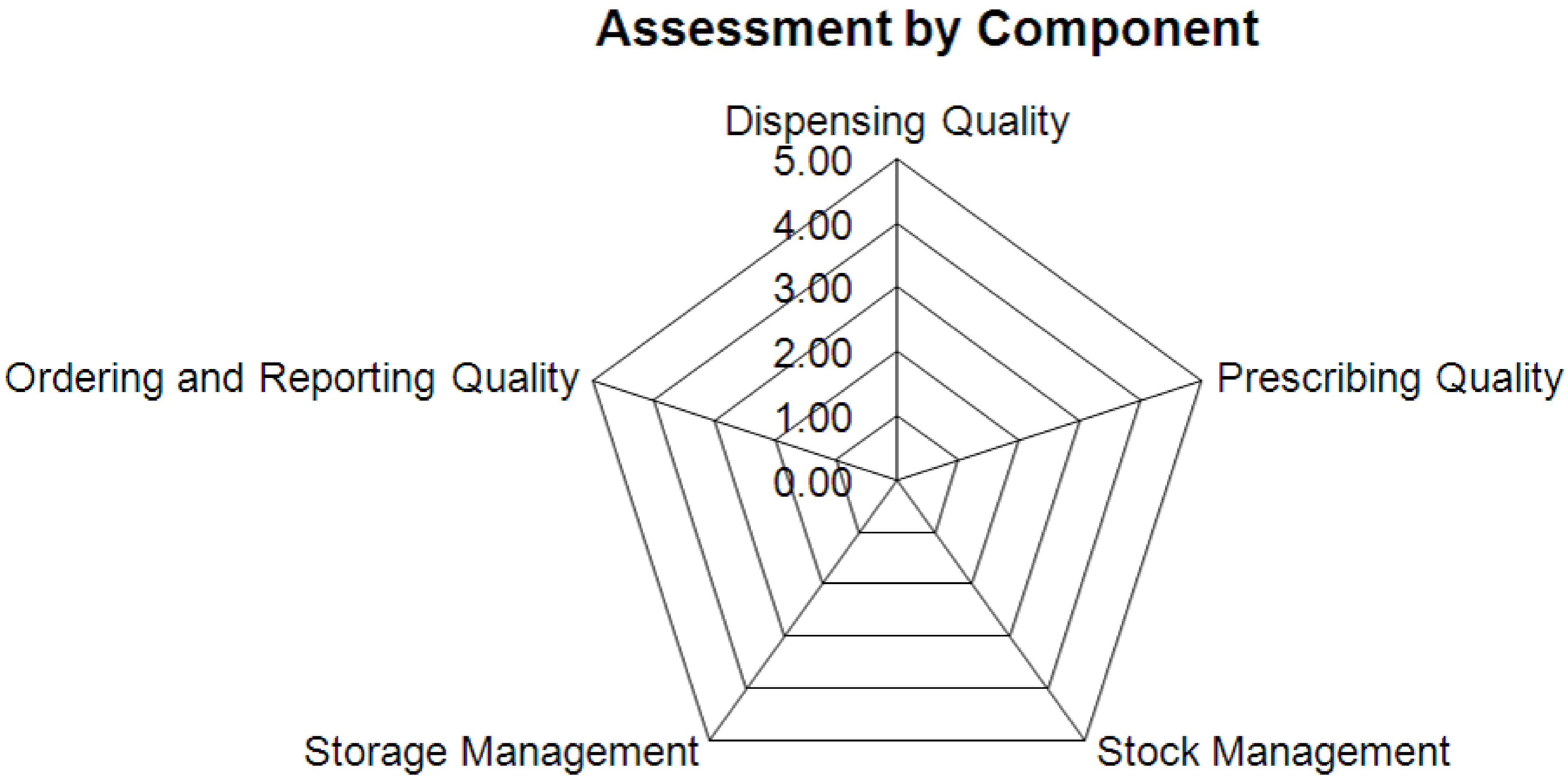

Assessment by Component
Dispensing Quality
5.00
4.00
3.00
2.00
1.00
0.00
Prescribing Quality
Stock Management
Storage Management
Ordering and Reporting Quality
