# Supplementary material for: Article 4: Impact assessment of supervision performance assessment and recognition strategy (SPARS) to improve supply chain management in health facilities in Uganda: a national pre and post study
Source: J Pharm Policy Pract. 2021 Feb 4;14:14. doi: 10.1186/s40545-020-00290-8 (PMC7857862; doi:10.1186/s40545-020-00290-8)
Supplement: Supplementary file 2 — Additional file 2: Supply chain management Assessment Indicators and measures. [file 40545_2020_290_MOESM2_ESM.pdf]

Additional file 2: Supply chain management Assessment Indicators and measures

SCM Assessment Indicators and Measures
**Stock management**
13. Stock card availability
14. Is stock card filled correctly?
15. Does stock card balance and recorded count of medicines in stock agree?
**Measures for storage management**
**17. Cleanliness of the pharmacy and store**
a) The pharmacy is acceptably clean
b) The main store is acceptably clean
**18. Hygiene of the pharmacy**
a) Are toilet facilities available?
b) Are the toilet facilities acceptable, hygienic and functioning?
c) Is there toilet paper?
d) Are hand-washing facilities available?
e) Are hand-washing facilities acceptable, hygienic and functioning?
f) Is there soap for hand washing?
**19. System for storage of medicines and health supplies**
a) Are medicines stored only on shelves, in cupboards, or on pallets, not directly on the floor?
b) Are medicines stored on shelves or in cupboards stored in a systematic manner (alphabetic, therapeutic, formulations)?
c) Are the shelves labelled?
***20*. Storage conditions**
a) Are there **NO** signs of pests/harmful insects/rodents seen in the area (Check traces, droppings etc. from bats, rats etc.)
b) Are the medicines protected from direct sunlight (painted glass, curtains, blinds or no windows?
c) Is the temperature of the storage room monitored and recorded daily?
d) Can the temperature of the storeroom be regulated (with ventilation, air-condition or by opening windows)?
e) Roof is maintained in good condition to avoid water penetration?
f) Is storage space meeting requirements (pharmacy > $4m^2$; store HC2/HC3 > $6m^2$; store HC4/Hosp. > $20m^2$)?
g) Is the pharmacy/store lockable and access limited to authorised personnel?
h) Is functional fire safety equipment (fire extinguisher, bucket with sand, water, or blanket) available and accessible?
i) Is there a functioning system for cold storage for vaccines (refrigerator)?
j) If yes, are only medicines stored in the refrigerator – no food or beverages?
k) Are vaccines placed in the center of refrigerator (not in the door)?
l) Is the refrigerator temperature recorded?
**21. Storage practices of medicines in the pharmacy (stores & dispensary)**
**Store**
a) Boxes are not directly on the floor in the store?
b) Is there a record for expired drugs (Check)?
c) Is there a place to store expired medicine separately? (Box clearly marked expired or in a shelf)

d) Is FEFO adhered to? (Check 20 randomly selected medicines)
**Dispensary**
e) Are opened bottles/ tins labelled with the opening date?
f) Do all tins/bottles that have been opened have a lid on?
**Measures for ordering and reporting**
**22. Reorder level calculation**
**23. Filing:** Are the following filed? Check and see
a) Discrepancy reports
b) Delivery notes
24. Do the HMIS report and stock card agree?

*(Assume shelves/ cupboard/pallets are available)

** (Assumes availability of a monitoring book)
