# Supplementary material for: Article 4: Impact assessment of supervision performance assessment and recognition strategy (SPARS) to improve supply chain management in health facilities in Uganda: a national pre and post study
Source: J Pharm Policy Pract. 2021 Feb 4;14:14. doi: 10.1186/s40545-020-00290-8 (PMC7857862; doi:10.1186/s40545-020-00290-8)
Supplement: Supplementary file 3 — Additional file 3: Completeness of measures at first and last visit. [file 40545_2020_290_MOESM3_ESM.pdf]

Additional file 3: Completeness of measures at first and last visit

SCM measure (n=1222)	Type of measure*	Completeness first visit (%)	Completeness last visit (%)	Change in completeness (%)	Completeness for both visits (%)
**Stock Management**					
Is stock card/ledger book available? (%)**	B	84%	80%	-4%	77%
Is stock card filled correctly? (%)**	B	88%	87%	-1%	78%
Does stock card balance and recorded count of medicines in stock agree? (%) **	B	85%	87%	2%	75%
**Storage Management**					
The dispensary is: Very clean & tidy (score=1) or Not clean/ untidy (score=0)	B	99%	92%	-7%	91%
The main store is: Very clean & tidy (score=1) or Not clean/ untidy (score=0)	B	99%	92%	-7%	91%
Are toilet facilities available?	R	99%	92%	-7%	91%
Are the toilet facilities acceptable, hygienic and functioning? (1/0)	B	89%	89%	0%	80%
Is there toilet paper?(1/0)	R	89%	88%	-1%	80%
Are hand washing facilities acceptable, hygienic and functioning? (1/0)	B	99%	92%	-7%	91%
Is there soap for hand washing? (1/0)	R	99%	92%	-7%	91%
Are medicines stored on shelves and /or in cupboards? (1/0)	R	99%	92%	-7%	91%
Are medicines stored on shelves or in cupboards stored in a systematic manner? (1/0)	B	99%	92%	-7%	91%
Are the shelves labelled?(1/0)	B	99%	92%	-7%	91%
If no signs of pests/harmful insects/rodents seen in the area? (1/0)	B	98%	92%	-6%	90%
Are the medicines protected from direct sunlight? (1/0)	B	98%	92%	-6%	90%
Is the temperature of the storage room monitored? (1/0)	B	98%	91%	-7%	90%
Can the temperature of the storeroom be regulated? (1/0)	R	98%	92%	-6%	90%
Roof is maintained in good condition to avoid water penetration? (1/0)	R	98%	92%	-6%	90%
Is storage space sufficient and adequate? (1/0)	R	98%	91%	-6%	89%
Is the store room lockable and access limited to authorized personnel? (1/0)	B	98%	92%	-6%	90%

SCM measure (n=1222)	Type of measure*	Completeness first visit (%)	Completeness last visit (%)	Change in completeness (%)	Completeness for both visits (%)
Fire safety equipment is available and accessible? (1/0)	B	98%	92%	-6%	90%
Is there a functioning system for cold storage (Refrigerator)? (1/0)	R	93%	88%	-5%	82%
If yes, are only medicines stored in the refrigerator – no food or beverage? (1/0)	B	66%	67%	1%	56%
Are vaccines placed in the center of refrigerator (not in the door)? (1/0)	B	66%	66%	1%	56%
Is the temperature of the refrigerator recorded? (1/0)	B	66%	66%	0%	56%
Boxes are not directly on the floor in the store? (1/0)	B	99%	92%	-7%	91%
Is there a record for expired drugs (Check)? (1/0)	B	99%	92%	-7%	91%
Is there a place to store expired medicine separately? (1/0)	B	99%	92%	-7%	91%
Is FEFO adhered to? (Check 20 randomly selected medicines if all are adherent to FEFO score yes else no)	B	99%	92%	-7%	91%
Are opened bottles labelled with the opening date? (1/0)	B	99%	92%	-7%	91%
Do all tins/bottles that have been opened have a lid on (dispensary)? (1/0)	B	99%	92%	-7%	91%
**Ordering and Reporting**					
Reorder level calculation. If correct yes (1), else no (0).	B	88%	89%	1%	79%
Do the HMIS report and stock card agree*** %	B	43%	61%	18%	31%
Filing of delivery notes done? (1/0)	B	94%	88%	-5%	83%
Filing of discrepancy reports done? (1/0)	B	92%	86%	-7%	80%
**Overall Average**		**86%**	**82%**	**-3%**	**77%**

* B=behavioral dependent; R=resource dependent; ** Average across up eight items measured; *** Average across six items measured
