# Supplementary material for: Article 4: Impact assessment of supervision performance assessment and recognition strategy (SPARS) to improve supply chain management in health facilities in Uganda: a national pre and post study
Source: J Pharm Policy Pract. 2021 Feb 4;14:14. doi: 10.1186/s40545-020-00290-8 (PMC7857862; doi:10.1186/s40545-020-00290-8)
Supplement: Supplementary file 4 — Additional file 4: Facility and visit characteristics. [file 40545_2020_290_MOESM4_ESM.pdf]

Additional file 4: Facility and visit characteristics

Study facilities	Total		HC2		HC3		HC4/hospital		χ^2
	No.	%	No.	%	No.	%	No.	%	p-value
	1222	100	681	100	416	100	125	100	
**Region**									
Central	250	21	133	20	92	22	25	20	0.343
Western	421	35	224	33	145	35	52	42	
Eastern	379	31	226	33	118	28	35	28	
Northern	172	14	98	14	61	15	13	10	
**Ownership**									
Government	1039	85	596	88	349	84	94	75	0.002
PNFP	183	15	85	13	67	16	31	25	
**Year of initial visit**									
2011	753	62	368	54	289	70	96	77	<0.001
2012	406	33	263	39	117	28	26	21	
2013	63	5	50	7	10	2	3	2	
**Health workers supervised at initial visit**									
One	280	23	223	33	45	11	12	10	<0.001
More than one	942	77	458	67	371	89	113	90	
**MMS supervising during initial visit**									
One	957	78	603	89	292	70	62	50	<0.001
More than one	265	22	78	12	124	30	63	50	
**Designated MMS supervised initial visit***									
No	394	32	208	31	150	36	36	29	0.118
Yes	828	68	473	69	266	64	89	71	

*Designated MMS is the MMS assigned to a facility who was responsible for a majority of visits
