# Supplementary material for: Article 4: Impact assessment of supervision performance assessment and recognition strategy (SPARS) to improve supply chain management in health facilities in Uganda: a national pre and post study
Source: J Pharm Policy Pract. 2021 Feb 4;14:14. doi: 10.1186/s40545-020-00290-8 (PMC7857862; doi:10.1186/s40545-020-00290-8)
Supplement: Supplementary file 5 — Additional file 5: Medicines management supervisor and district health officer characteristics. [file 40545_2020_290_MOESM5_ESM.pdf]

## Additional file 5: Medicines management supervisor and district health officer characteristics

| Characteristics | No. | % |
|---|---|---|
| **MMS study total** | **148** | **100** |
| **Gender** | | |
| Male | 124 | 84 |
| Female | 24 | 16 |
| **Level** | | |
| District MMS | 53 | 36 |
| Sub district MMS | 95 | 64 |
| **Regions** | | |
| Central | 31 | 21.0 |
| Western | 56 | 37.8 |
| Eastern | 41 | 27.7 |
| Northern | 20 | 13.5 |
| **Facilities supervised** | | |
| 1-10 | 81 | 54.7 |
| 11-15 | 47 | 31.7 |
| 16+ | 20 | 13.6 |
| **Professional training** | | |
| Clinical officer | 87 | 59 |
| Pharmacist/dispenser | 15 | 10 |
| Nurse | 36 | 24 |
| Supplies officer | 10 | 7 |
| **MMS completing 2013 survey** | 111 | 75 |
| **Age group** | | |
| 26-35 | 37 | 34 |
| 36-45 | 46 | 42 |
| 46+ | 26 | 24 |
| **Highest level of education** | | |
| Secondary/diploma/other | 92 | 83 |
| Bachelors/Master's degree | 19 | 17 |
| **Number of years of work experience** | | |
| 0-9 | 45 | 40 |
| 10+ | 66 | 60 |
| **Frequency of meetings with DHO** | | |
| Monthly/weekly | 60 | 54 |
| Quarterly/semi-annually | 24 | 22 |
| Irregularly/other | 27 | 24 |
| **Received feedback from DHO about MMS report** | | |
| No | 16 | 15 |
| Yes | 92 | 85 |
| **Sufficient time during visits to provide adequate supportive supervision** | | |
| No | 38 | 35 |
| Yes | 71 | 65 |
| **Health workers respond well to supervision** | | |
| Some of them | 40 | 37 |
| Most/all of them | 68 | 63 |
