# Supplementary material for: Article 4: Impact assessment of supervision performance assessment and recognition strategy (SPARS) to improve supply chain management in health facilities in Uganda: a national pre and post study
Source: J Pharm Policy Pract. 2021 Feb 4;14:14. doi: 10.1186/s40545-020-00290-8 (PMC7857862; doi:10.1186/s40545-020-00290-8)
Supplement: Supplementary file 6 — Additional file 6: Number of MMS visits within the first year of SPARS supervision, overall and by level of care. [file 40545_2020_290_MOESM6_ESM.pdf]

Additional file 6: Number of MMS visits within the first year of SPARS supervision, overall and by level of care

No. of visits	All facilities		HC2		HC3		HC4/hospitals	
	No.	%	No.	%	No.	%	No.	%
2	328	27	184	27	115	28	29	23
3	334	27	176	26	115	28	43	34
4	323	26	180	26	108	26	35	28
5	201	16	122	18	62	15	17	14
6	35	3	19	3	15	4	1	1
7	1	0	0	0	1	0	0	0
**Total**	**1222**	**100**	**681**	**100**	**416**	**100**	**125**	**100**
